# Knowledge and beliefs about Ebola virus in a conflict-affected area: early evidence from the North Kivu outbreak

**DOI:** 10.7189/jogh.09.020311

**Published:** 2019-12

**Authors:** Ben Oppenheim, Nicholai Lidow, Patrick Ayscue, Karen Saylors, Placide Mbala, Charles Kumakamba, Michael Kleinman

**Affiliations:** 1 Metabiota, San Francisco, California, USA; 2 Orange Door Research, San Francisco, California, USA; 3 Institut National de Recherche Biomédicale (INRB), Kinshasa, Democratic Republic of the Congo; 4 Metabiota, Kinshasa, Democratic Republic of the Congo

The current Ebola virus disease (EVD) outbreak in the Democratic Republic of the Congo (DRC) – the country’s 10^th^ – is centered in North Kivu, a region affected by long-running armed conflict. As of September 15 2019, 3129 cases have been reported as well as 2096 deaths, giving an estimated case fatality ratio of approximately 67% [1].

North Kivu presents a particularly challenging context for outbreak control. Conflict and population displacement are well-known to facilitate disease transmission and inhibit humanitarian response efforts [2]. Estimates suggest that approximately 319,000 people were displaced from North Kivu and Ituri by violence during January - February 2018 [3], and although displacement data are not systematically available, conflict-induced forced migration has continued. Ongoing insecurity across the outbreak area threatens the ability of health workers to investigate cases, trace contacts, deliver care, and administer vaccines.

The World Health Organization (WHO) has stated [4] that health workers in the affected area are regularly accompanied by armed Civil Protection escorts when deployed to “red zones” designated as high security risk, particularly when they are searching for unidentified case contacts. It is essential that health workers be adequately protected. However, the blending of military and health deployments can raise hazards to medical personnel [5], particularly if health workers are perceived to have lost impartiality.

Given the history of violence perpetrated against civilian populations in North Kivu by armed groups, including elements of the armed forces [6], there are tensions between the government and civilian populations, and as a result, potential risks to co-deployments. These tensions are not unique to North Kivu. Benton [7] notes that military involvement in outbreak response can – regardless of intent – signal the potential for coercive control over civilian populations, for example forced compliance, or movement controls among affected communities. The potential for communities to interpret a military presence in coercive terms is potentially greater in a region like North Kivu, and has implications for public attitudes, behaviors and reactions to disease control efforts.

Unfortunately, there are limited data on public perceptions of the outbreak and sentiment towards health workers and the military within conflict-affected areas of the DRC (although recent research has started to address these issues [8]). To gather data on public trust and perceptions towards health workers and other responders, we initiated a survey within two weeks of the official declaration of the outbreak on August 1st, 2018. [9]

Survey respondents were selected via simple random sampling from a panel of potential respondents across the Orange and Vodacom Networks in North Kivu, who had earlier agreed to participate in surveys through the GeoPoll platform. The GeoPoll panel was constructed from lists of cell phone subscribers in North Kivu provided by mobile phone operators and is updated on a quarterly basis; the panel for North Kivu consists of approximately 1 180 000 potential respondents.

We used an SMS survey for two reasons: first, in-person surveys (particularly those in conflict-affected areas) generally have some margin of start-up time given the need to train and deploy enumerators, and we aimed to generate survey data as close to the onset of the outbreak as possible. Second, while SMS surveys have limitations which should be borne in mind (discussed below), they allow for inexpensive, safe enumeration of a wide geographic area, potentially reaching populations that are difficult to access amidst unstable conditions.

**Figure:**
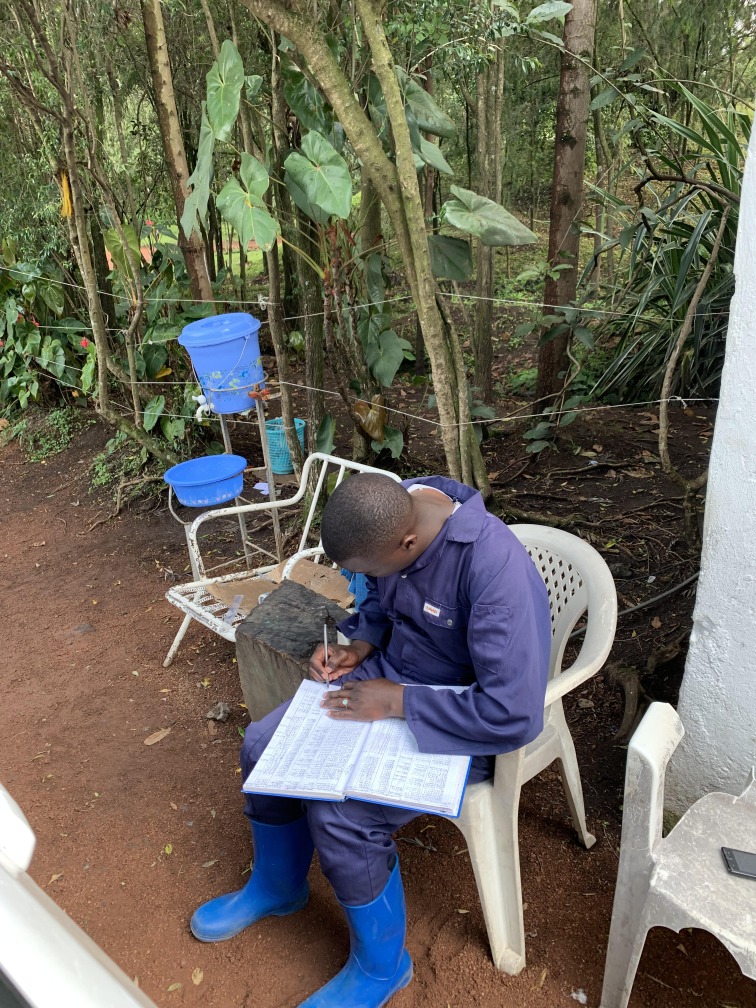
Photo: Health worker recording the temperature of people entering the Ebola Coordination Center in Goma, DRC (from the collection of Irene L. Stephan, used with permission).

The survey collected data on awareness of the outbreak, local beliefs regarding the cause of Ebola’s spread within North Kivu, trust in health workers, and willingness to be vaccinated. The survey also collected demographic data (gender, age) as well as the respondent’s report of their geographic location, and used iterative proportional fitting to generate survey weights by province based on gender, age group, and geographic region that mirror the demographics of the province (for more information on the survey, questionnaire and weighting, see Appendices S1 and S2 in **Online Supplementary Document**) [10]. 658 respondents completed the survey between August 17th and September 11th. Descriptive statistics reported below were estimated from demographically weighted responses.

We found a high level of awareness of the current outbreak across North Kivu, with 92% (95% confidence interval CI = 89%-95%) of respondents reporting that they were aware. 83% (95% CI = 80%-87%) of respondents in North Kivu indicate trusting health workers, and 85% (95% CI = 82%-89%) stated willingness to receive a vaccine against Ebola virus. The estimates of public awareness of the outbreak are similar to estimates produced by Vinck et al. in a population survey conducted between September 1 and 16, which found 100 percent awareness among respondents. However, estimates of trust in health workers and willingness to take the Ebola vaccine by Vinck et al. were substantially lower at 61.5 percent and 63.3 percent, respectively (**Table 1**) [8].

**Table 1:** Public knowledge and attitudes regarding Ebola virus and responders, percent of population (95% confidence intervals)

	Total	Region*		
**Goma (extended)**	**Grand nord**	**Petit nord**
**Perceptions of how Ebola virus is spread:**				
Perceived transmission route:				
Bats	11 (8-13)	10 (6-13)	12 (7-18)	11 (5-17)
Bushmeat	32 (28-36)	32 (27-38)	32 (25-40)	27 (19-36)
Equateur outbreak	14 (14-17)	15 (11-19)	11 (6-16)	16 (9-23)
Health workers	2 (1-3)	2 (0-3)	2 (0-4)	4 (0-8)
Infected people	26 (22-30)	27 (22-32)	23 (16-30)	20 (13-28)
FARDC	2 (1-3)	2 (0-3)	1 (0-2)	5 (1-9)
Witchcraft	2 (1-3)	2 (0-3)	3 (0-5)	5 (1-10)
Other	12 (9-15)	11 (7-14)	17 (11-23)	11 (5-17)
**Trust in health workers and willingness to take an Ebola vaccine:**				
Trust health workers	83 (80-87)	82 (77-86)	89 (83-94)	86 (79-93)
Willing to take vaccine	85 (82-89)	84 (80-89)	89 (84-94)	84 (77-91)
N	658	366	166	126

FARDC – Armed Forces of the Democratic Republic of the Congo

* Goma (extended) includes the Goma municipal area as well as the neighboring territory of Nyiragongo. Grand nord includes the territories Beni, Butembo, Lubero, and Oicha. Petit nord includes the remaining territories of Masisi, Rutshuru, and Walikale.

Differences in geographical coverage are likely to explain some of the differences in estimates, as the Vinck et a. study enumerated urban households in Beni and Butembo, while ours covered a broader geographic area. Generally speaking, trust in cities in the region is higher than in rural areas (see for example survey estimates for DRC published by Vinck and others at http://www.peacebuildingdata.org/). It is also possible that the mobile phone survey produced a sample with higher levels of education and income than the Vinck et al. household survey. And indeed, in the Vinck et al. data, trust in health workers and vaccine acceptance are correlated with education. For example, 56% of respondents with incomplete primary education reported trusting health workers, compared to 67.1% among those with completed secondary education; 56.9% of respondents with incomplete primary education reported willingness to take a vaccine against Ebola, compared to 70.9% of respondents with complete secondary education [11].

Similar to prior evidence from Sierra Leone [12], we find that the population in North Kivu views consuming bushmeat as a primary vector for the spread of Ebola (21%, 95% CI = 28%-36%), followed by contact with infected people (26%, 95% CI = 22%-30%). We also found evidence that a small proportion of the population believes that the Ebola virus is being spread by the Armed Forces of the DRC (the FARDC) (2%, 95% CI = 1%-3%), and by health workers (2%, 95% CI = 1%-3%). When we spatially disaggregated the data to the sub-regional level, we found indications that such beliefs may be distributed unevenly across localities.

Sub-regional estimates are less precise than provincial estimates, owing to sample size limitations, but suggest potentially important spatial variation in public knowledge and attitudes. Beliefs about the Congolese military spreading Ebola appear to be highest in Petit nord (5%, 95% CI = 1%-9%), an area that has seen intense conflict and violence against civilian populations. Estimates of public belief that health workers are spreading the virus were also highest in Petit nord (4%, 95% CI = 0%-8%). These estimates should be interpreted with caution, due to overlapping confidence intervals across the sub-regions, but indicate that public knowledge and attitudes in Petit nord and beyond warrant further investigation to inform risk assessments to surveillance and vaccination teams. More generally, in an area with complex localized violence and political instability like North Kivu, public knowledge and attitudes should be assessed at the sub-regional level.

It should be noted that collecting data via SMS messaging necessarily means that segments of the population with little or no literacy will be excluded. It is possible that exposure to (and belief in) outbreak-related rumors and misinformation could vary by education level and therefore bias the estimates we discuss here, though a prior multi-country study of rumors in conflict areas found no consistent relationship between education level and belief in rumors [13].

While there is a growing body of evidence on public knowledge, attitudes and practices with respect to Ebola virus [12], there is limited data on areas like North Kivu, which are affected by ongoing violence and deep distrust towards government institutions. Because Ebola’s estimated ecological niche includes a number of countries and sub-regions affected by long-running violence and public mistrust of government [14], it is important to develop evidence to support an effective and safe public health response, and carefully calibrate strategies to protect health workers so as to limit the risk of exacerbating tensions.

Since we conducted our survey, the North Kivu outbreak has continued unabated, driven by a steady case count, challenges working within affected communities to trace and treat contacts, and sporadic but serious violence against treatment centers and health workers.

The relatively small sample size means that the geographically-disaggregated estimates we discuss here come with uncertainty, particularly for low-prevalence beliefs like military forces spreading Ebola. Nonetheless, beliefs regarding emergency responders in a conflict-affected area like North Kivu have direct implications for the tenor of community engagement with health workers, as well as their security and their effectiveness in outbreak surveillance and containment, and we believe warrant further investigation.

Timely data on public knowledge, attitudes and beliefs can help inform resource and deployment decisions, shed light on the benefits and potential risks of deployment to particular localities, and inform decisions to engage military forces to provide operational security. More granular information on localized beliefs can potentially help mitigate risk to health workers, and identify potential consequences of security strategies like military accompaniment. In addition, longitudinal data on public perceptions may be useful in mapping changing awareness, the impact of risk communication campaigns, and shifting patterns of trust in health and security personnel, as the epidemic and public health response unfold.

## Additional material

Online Supplementary Document

## References

[1] World Health Organization. Ebola Virus Disease: Democratic Republic of the Congo. External Situation Report No 59. Available: https://apps.who.int/iris/rest/bitstreams/1248907/retrieve. Accessed: 21 September 2019.

[2] Gayer M Legros D Formenty P Connolly MA Conflict and emerging infectious diseases. Emerg Infect Dis. 2007;13:1625. 10.3201/eid1311.061093 18217543 PMC3375795

[3] United Nations Office for the Coordination of Humanitarian Affairs. Plan de Réponse d’Urgence: Provinces du Nord-Kivu et de L’Ituri (RD Congo); 2018 https://reliefweb.int/sites/reliefweb.int/files/resources/rd_congo_-_provinces_nord-kivu_et_ituri_-_plan_de_reponse_durgence_avril-septembre_2018_0.pdf. Accessed 28 August 2019.

[4] World Health Organization Épidémie de la Maladie à Virus Ebola, Provinces du Nord-Kivu et de l’Ituri, République Démocratique du Congo. Rapport de Situation No. 2018;41:27.

[5] Inter-agency Standing Committee. Guidelines on the Use of Armed Escorts for Humanitarian Convoys; 2013. Available: https://www.unocha.org/sites/unocha/files/Armed%20Escort%20Guidelines%20-%20Final_1.pdf. Accessed; Accessed: 28 August 2019.

[6] Congo Research Group. Who Are the Killers of Beni? Investigative Report No. 1.; 2016. Available: http://congoresearchgroup.org/wp-content/uploads/2016/03/Rapport-Beni-GEC-21-mars.pdf. Accessed: 28 August 2019.

[7] Benton A. Whose security? Militarization and securitization during West Africa’s Ebola outbreak. In: Hofman M, Au S, eds. The Politics of Fear: Médecins sans Frontières and the West African Ebola Epidemic. New York: Barns & Noble; 2017. pp 25-50.

[8] Vinck P Pham PN Bindu KK Bedford J Nilles EJ Institutional trust and misinformation in the response to the 2018-19 Ebola outbreak in North Kivu, DR Congo: a population-based survey. Lancet Infect Dis. 2019;19:529-36. 10.1016/S1473-3099(19)30063-5 30928435

[9] Public Attitudes in North Kivu. Early Evidence from the DRC’s 10th Ebola Outbreak. Orange Door Research Working Paper No. 1. [unpublished manuscript]

[10] Leo B, Morello R, Mellon J, Peixoto T, Davenport ST. Do mobile phone surveys work in poor countries? Center for Global Development Working Paper. 2015 Apr 7(398)

[11] Personal correspondence with Patrick Vinck.

[12] Jalloh MF Sengeh P Monasch R Jalloh MB DeLuca N Dyson M National survey of Ebola-related knowledge, attitudes and practices before the outbreak peak in Sierra Leone: August 2014. BMJ Glob Health. 2017;2:e000285. 10.1136/bmjgh-2017-000285 29259820 PMC5728302

[13] Greenhill KM Oppenheim B Rumor has it: The adoption of unverified information in conflict zones. Int Stud Q. 2017;61:660-76. 10.1093/isq/sqx015

[14] Pigott DM Golding N Mylne A Huang Z Henry AJ Weiss DJ Mapping the zoonotic niche of Ebola virus disease in Africa. eLife. 2014;3:e04395. 10.7554/eLife.04395 25201877 PMC4166725

