# Supplementary material for: Knowledge and beliefs about Ebola virus in a conflict-affected area: early evidence from the North Kivu outbreak
Source: J Glob Health. 2019 Oct 22;9(2):020311. doi: 10.7189/jogh.09.020311 (PMC6812978; doi:10.7189/jogh.09.020311)
Supplement: Online Supplementary Document [file jogh-09-020311-s001.pdf]

Knowledge and Beliefs about Ebola Virus in a Conflict-Affected Area: Early Evidence from the North Kivu Outbreak

Appendix S1: Weighting

The most recent census in Democratic Republic of the Congo was conducted in 1984, and current demographic are based on a variety of estimates that come with inherent uncertainty, therefore complicating the generation of weights. For this study, a sex ratio of 100 females and 99 males was employed. Age groups were weighted roughly equally to match the country's overall age structure, with 33% of the survey weight assigned to respondents aged 15-24, 34% to respondents aged 25-34, and 33% to respondents aged 35 years or more. The extended Goma region was assumed to have 34% of the estimated 6 million population, while the Grand nord and Petit nord are assumed to have 28% and 38%, respectively. These estimates are based on data collected by the *Institut National de la Statistique* of the Democratic Republic of the Congo and published in their report, *Enquête EDS-RDC II 2013-2014.* The results presented in this paper are not sensitive to these assumptions. Weights and estimates were generated using the survey package in R. (See Lumley T. Complex surveys: a guide to analysis using R. John Wiley & Sons; 2011)

Appendix 2: Survey questions

Are you aware of any Ebola outbreaks currently happening in North Kivu?<br><br>1)Yes<br>2)No
If there was an Ebola outbreak in your community, would you trust outside health workers who came to find and treat sick people?<br><br>1) Trust Them<br>2) Don't Trust Them
Would you take a vaccine against Ebola if it was offered to you?<br><br>1)Yes<br>2)No

How do people in Kivu get Ebola?

1) Bushmeat
2) From infected people
3) From health workers
4) From the Equateur outbreak
5) Witchcraft
6) Spread by FARDC
7) Bats
8) Other
